# Exploring uncatalogued genetic variation in antimicrobial resistance gene families in *Escherichia coli*: an observational analysis

**DOI:** 10.1016/S2666-5247(24)00152-6

**Published:** 2024-10-05

**Authors:** Samuel Lipworth, Derrick Crook, A Sarah Walker, Tim Peto, Nicole Stoesser

**Affiliations:** Nuffield Department of Medicine, University of Oxford, Oxford, UK; Oxford University Hospitals NHS Foundation Trust, Oxford, UK; Nuffield Department of Medicine, University of Oxford, Oxford, UK; Oxford University Hospitals NHS Foundation Trust, Oxford, UK; NIHR Oxford Biomedical Research Centre, Oxford University Hospitals NHS Foundation Trust, John Radcliffe Hospital, Oxford, UK; NIHR Health Protection Research Unit in Healthcare Associated Infections and Antimicrobial Resistance at University of Oxford in partnership with UKHSA, Oxford, UK; Nuffield Department of Medicine, University of Oxford, Oxford, UK; NIHR Oxford Biomedical Research Centre, Oxford University Hospitals NHS Foundation Trust, John Radcliffe Hospital, Oxford, UK; NIHR Health Protection Research Unit in Healthcare Associated Infections and Antimicrobial Resistance at University of Oxford in partnership with UKHSA, Oxford, UK; Nuffield Department of Medicine, University of Oxford, Oxford, UK; Oxford University Hospitals NHS Foundation Trust, Oxford, UK; NIHR Oxford Biomedical Research Centre, UK; Oxford University Hospitals NHS Foundation Trust, John Radcliffe Hospital, Oxford, UK; Nuffield Department of Medicine, University of Oxford, Oxford, UK; Oxford University Hospitals NHS Foundation Trust, Oxford, UK; NIHR Oxford Biomedical Research Centre, Oxford University Hospitals NHS Foundation Trust, John Radcliffe Hospital, Oxford, UK; NIHR Health Protection Research Unit in Healthcare Associated Infections and Antimicrobial Resistance at University of Oxford in partnership with UKHSA, Oxford, UK

## Abstract

**Background:**

Antimicrobial resistance (AMR) in *Escherichia coli* is a global problem associated with substantial morbidity and mortality. AMR-associated genes are typically annotated based on similarity to variants in a curated reference database, with the implicit assumption that uncatalogued genetic variation within these is phenotypically unimportant. In this study, we evaluated the performance of the AMRFinder tool and, subsequently, the potential for discovering new AMR-associated gene families and characterising variation within existing ones to improve genotype-to-susceptibility phenotype predictions in *E coli*.

**Methods:**

In this cross-sectional study of international genome sequence data, we assembled a global dataset of 9001 *E coli* sequences from five publicly available data collections predominantly deriving from human bloodstream infections from: Norway, Oxfordshire (UK), Thailand, the UK, and Sweden. 8555 of these sequences had linked antibiotic susceptibility data. Raw reads were assembled using Shovill and AMR genes (relevant to amoxicillin–clavulanic acid, ampicillin, ceftriaxone, cipro-floxacin, gentamicin, piperacillin–tazobactam, and trimethoprim) extracted using the National Center for Biotechnology Information AMRFinder tool (using both default and strict [100%] coverage and identity filters). We assessed the predictive value of the presence of these genes for predicting resistance or susceptibility against US Food and Drug Administration thresholds for major and very major errors. Mash was used to calculate the similarity between extracted genes using Jaccard distances. We empirically reclustered extracted gene sequences into AMR-associated gene families (≥70% match) and antibiotic-resistance genes (ARGs; 100% match) and categorised these according to their frequency in the dataset. Accumulation curves were simulated and correlations between gene frequency in the Oxfordshire and other datasets calculated using the Spearman coefficient. Firth regression was used to model the association between the presence of *bla*_TEM-1_ variants and amoxicillin–clavulanic acid or piperacillin–tazobactam resistance, adjusted for the presence of other relevant ARGs.

**Findings:**

The performance of the AMRFinder database for genotype-to-phenotype predictions using strict 100% identity and coverage thresholds did not meet US Food and Drug Administration thresholds for any of the seven antibiotics evaluated. Relaxing filters to default settings improved sensitivity with a specificity cost. For all antibiotics, most explainable resistance was associated with the presence of a small number of genes. There was a proportion of resistance that could not be explained by known ARGs; this ranged from 75⋅1% for amoxicillin–clavulanic acid to 3⋅4% for ciprofloxacin. Only 18 199 (51⋅5%) of the 35 343 ARGs detected had a 100% identity and coverage match in the AMRFinder database. After empirically reclassifying genes at 100% nucleotide sequence identity, we identified 1042 unique ARGs, of which 126 (12⋅1%) were present ten times or more, 313 (30⋅0%) were present between two and nine times, and 603 (57⋅9%) were present only once. Simulated accumulation curves revealed that discovery of new (100% match) ARGs present more than once in the dataset plateaued relatively quickly, whereas new singleton ARGs were discovered even after many thousands of isolates had been included. We identified a strong correlation (Spearman coefficient 0⋅76 [95% CI 0⋅73–0⋅80], p<0⋅0001) between the number of times an ARG was observed in Oxfordshire and the number of times it was seen internationally, with ARGs that were observed six times in Oxfordshire always being found elsewhere. Finally, using the example of *bla*_TEM-1_, we showed that uncatalogued variation, including synonymous variation, is associated with potentially important phenotypic differences; for example, two common, uncatalogued *bla*_TEM-1_ alleles with only synonymous mutations compared with the known reference were associated with reduced resistance to amoxicillin–clavulanic acid (adjusted odds ratio 0⋅58 [95% CI 0⋅35–0⋅95], p=0⋅031) and piperacillin–tazobactam (0⋅50 [95% CI 0⋅29–0⋅82], p=0⋅005).

**Interpretation:**

We highlight substantial uncatalogued genetic variation with respect to known ARGs, although a relatively small proportion of these alleles are repeatedly observed in a large international dataset suggesting strong selection pressures. The current approach of using fuzzy matching for ARG detection, ignoring the unknown effects of uncatalogued variation, is unlikely to be acceptable for future clinical deployment. The association of synonymous mutations with potentially important phenotypic differences suggests that relying solely on amino acid-based gene detection to predict resistance is unlikely to be sufficient. Finally, the inability to explain all resistance using existing knowledge highlights the importance of new target gene discovery.

**Funding:**

National Institute for Health and Care Research, Wellcome, and UK Medical Research Council.

## Introduction

Antimicrobial resistance (AMR) is a global challenge with substantial associated morbidity and mortality.^1^ In *Escherichia coli*, AMR is mostly conferred by acquisition of genes that can be integrated into the chromosome or carried on plasmids.^2–4^ AMR can also occur via point mutations in both core and accessory genes. Extensive efforts have been made to catalogue and characterise these mechanisms, resulting in several highly curated databases that are widely used for genomic epidemiology.^5–7^

Several studies have investigated the performance of such databases to predict phenotype from genotype for *E coli*,^8,9^ highlighting the associated challenges. Verschuuren and colleagues^10^ recently showed the inability of the ResFinder tool to meet US Food and Drug Administration (FDA) specifications for major or very major error rates for most antibiotics (234 isolates, selected for resistance to third-generation cephalosporins). This work found particularly poor performance in predicting AMR phenotype for β-lactam–β-lactamase inhibitor combination drugs, replicating a finding from earlier studies.^11,12^ Feldgarden and colleagues^6^ claimed much better performance (99⋅7% overall concordance when pooling across antibiotic classes), albeit with a small (47 isolates) and predominantly susceptible dataset. These and other studies highlight the need for further development and expansion of these databases if they are to become clinically useful.

There are two hierarchical levels of annotation within existing AMR gene catalogues: AMR-associated gene families (eg, *bla*_*CTX-M*_, *gyrA*) and alleles of gene families (eg, *gyrA* Ser83Leu, *bla*_*CTM-M-15*_, *bla*_*CTX-M-27*_); we hereafter refer to the latter as antibiotic resistance genes (ARGs). It is currently standard practice to characterise the presence or absence of ARGs based on percentage identity and coverage (commonly used thresholds for the former are 80% [the ABRicate default] and 90% [the AMRFinder default]).^13^ Where there is no perfect match, the presence of the closest characterised allele in the same gene family is reported; hence, most studies ignore any non-catalogued variation. To our knowledge, the prevalence, diversity, and impact of these imperfectly matching genes has not been systematically evaluated. Improvements to existing catalogues might, therefore, come from discovery of novel AMR-associated gene families or improved annotation of variation within existing ones.

In this study, we seek to estimate the potential for further exploration of these two domains of genomic variation to improve existing ARG reference databases. We first quantify how much resistance is explained by presence or absence of known ARGs or variants, and therefore estimate how much might be gained by searching for novel AMR-associated gene families. Second, we explore variation within known AMR-associated gene families at 100% match (acknowledging that this could be caused by sequencing or assembly error as well as true biological variation) that is currently uncatalogued and investigate whether including this in future versions of databases is likely to be useful.

## Methods

### Study design

In this international cross-sectional study, we selected five large *E coli* sequencing projects for inclusion, which were predominantly from human bloodstream infections: PRJEB11403 (Thailand, 2014–15, data unpublished), PRJEB23294 (various countries including Sweden, 2018),^12^ PRJEB32059 (Norway, 2002–17),^14^ PRJEB4681 (UK, 2001–11),^15^ and PRJNA604975 (Oxfordshire, UK, 2008–18).^16^ We selected these studies because they had linked whole-genome sequencing and antimicrobial susceptibility phenotype data available. Raw reads from isolates in these BioProjects were downloaded from the European Nucleotide Archive and subsequently assembled using Shovill (version 1.0.4^17^) using default settings. We excluded assemblies with total size less than 4 000 000 or more than 6 000 000, and those that did not have associated antimicrobial susceptibility data (all binary and measured using European Committee on Antimicrobial Susceptibility Testing breakpoints) for at least one antibiotic. A permanova was performed to explore whether there were any differences in the ARG content of these isolates (appendix 1 p 2). Quast (version 5.2.0)^18^ was used to generate assembly quality control metrics. As this study was a retrospective secondary analysis of publicly available data, no ethical approval was required.

### Procedures

We chose to focus on ARGs encoding resistance to seven drugs in five clinically relevant antibiotic classes (as defined by AMRFinder) for the treatment of *E coli* infection in humans: aminoglycosides (gentamicin), β-lactams (ampicillin and the β-lactamase inhibitor combinations amoxicillin–clavulanic acid and piperacillin–tazobactam), cephalosporins (ceftriaxone), quinolones (ciprofloxacin), and trimethoprim. We ran the AMRFinder software (v3.10.23,^19^ database version 2022-12-19.1) using the -O Escherichia –nucleotide output flags (using the default curated or 90% identity threshold and default 50% minimum coverage threshold). We extracted all sequences for each antibiotic class into a single multi-FASTA file, and then sketched these sequences (Mash^20^ sketch -s 100 000 -i) and created an all versus all distance matrix from the number of shared hashes divided by the total number of hashes.

Given that AMR gene nomenclature sometimes assigns similar gene namesto ARGs that are genetically diverse, and different gene names to ARGs that are genetically similar, we empirically redefined AMR-associated gene families and ARGs (appendix 1 p 8). We defined AMR-associated gene families by filtering mash distance matrices for any given antibiotic class at a minimum 0⋅7 similarity threshold (ie, 70% kmer of all possible kmers match exactly, approximately similar to the threshold used by Panaroo^21^ and others to define gene families). We performed no such reclassification for genes belonging to the point AMRFinder element subtype (eg, *gyrA, parC*) as these are core genes that are not difficult to accurately identify. These filtered distance matrices were then converted into graphs from which communities (AMR-associated gene families) were detected using complete linkage (R package igraph^22^). AMR-associated gene families were named according to the most common label assigned to their members by AMRFinder. We hereafter refer to each unique version (including the reference sequence or wild-type) of any AMR-associated gene family as an ARG, regardless of whether it contains one or more single-nucleotide polymorphisms or indels compared with the reference sequence. To define these ARGs, we repeated the process above with a 1⋅0 similarity threshold (ie, 100% sequence identity and coverage) and assigned sequential numeric labels to define unique ARGs within a given AMR-associated gene family (*bla*_TEM-1 1_, *bla*_TEM-1 2_, etc).

Statistical analysis

To determine how much resistance remains unexplained by existing ARG catalogues, we first quantified the extent to which the genes or mutations identified by the AMRFinder database were able to explain the observed resistance phenotype. Isolates were predicted as being resistant to an antibiotic if their genotype contained any allele associated with resistance to the drug in the AMRFinder database (this analysis was first performed with strict 100% coverage and identity filters and subsequently using the AMRFinder default settings [ie, 90% or curated identity threshold, 50% coverage] as well as with intermediate values in a sensitivity analysis for ciprofloxacin; appendix 1 p 4). Because AMRFinder does not provide any phenotypic subclassifications of β-lactam-resistance encoding ARGs, we used the lookup table provided by ResFinder^5^ to predict phenotypes for β-lactam–β-lactamase inhibitor combination drugs. Sensitivity, specificity, negative predictive value, and positive predictive value were calculated in the standard way (code provided in binder environment), making comparisons against the laboratory-derived antibiotic susceptibility phenotypes that were available for the sequences analysed as the reference standard. We also calculated the frequency of major errors (ie, erroneous genotypic prediction of susceptible isolates as resistant when compared with the reference phenotype) and very major errors (ie, erroneous genotypic prediction of resistant isolates as susceptible when compared with the reference phenotype). We used the FDA guidance on acceptable performance standards for these as a reference (major error <3%, upper confidence limit for very major error <7⋅5%).^23^ We estimated exact binomial 95% CIs using the R package Stats. We performed a sensitivity analysis to explore the extent to which the observed results might be affected by the prevalence of resistance in our dataset (appendix 1 p 2), and separate stratified analyses to investigate whether there was evidence of substantial heterogeneity in performance characteristics between studies (appendix 1 p 9).

We classified ARGs according to their overall frequency in the dataset (ie, occurring only once [singletons], between two and nine times, or ten times or more). To determine the rate at which new ARGs in these categories were discovered as more genomes in the dataset were analysed, rarefaction curves were created after randomisation of the isolate order using the rarefaction function of the R package Micropan (n.perm=100).^24^ To explore whether patterns of ARGs selection were similar between datasets from the studies included in this analysis, we calculated the correlation between the number of times an ARG was observed in the Oxfordshire versus other datasets using the Spearman correlation coefficient (R package Stats). Firth regression (R package logistf^25^) was used to investigate whether different 100% match variants of *bla*_TEM-1_ were associated with a higher probability of resistance to amoxicillin–clavulanic acid or piperacillin–tazobactam, including multivariable models to adjust for the presence of other ARGs known to cause resistance to these antibiotics. Firth regression was used to estimate odds ratios or adjusted odds ratios (aORs) for these associations due to the presence of *bla*_CMY-2_ being a perfect predictor for amoxicillin–clavulanic acid resistance, meaning standard logistic regression does not converge. An additional analysis was performed to explore the extent to which this association might be confounded by population structure (appendix 1 p 2). Finally, we investigated the extent to which sequencing or bioinformatic error might inflate the true number of ARGs that occurred only once (singletons; appendix 1 p 2). All statistical analyses were done with R version 4.3.1.

### Role of the funding source

The funders of the study had no role in study design, data collection, data analysis, data interpretation, or writing of the report.

## Results

**Figure 1:**
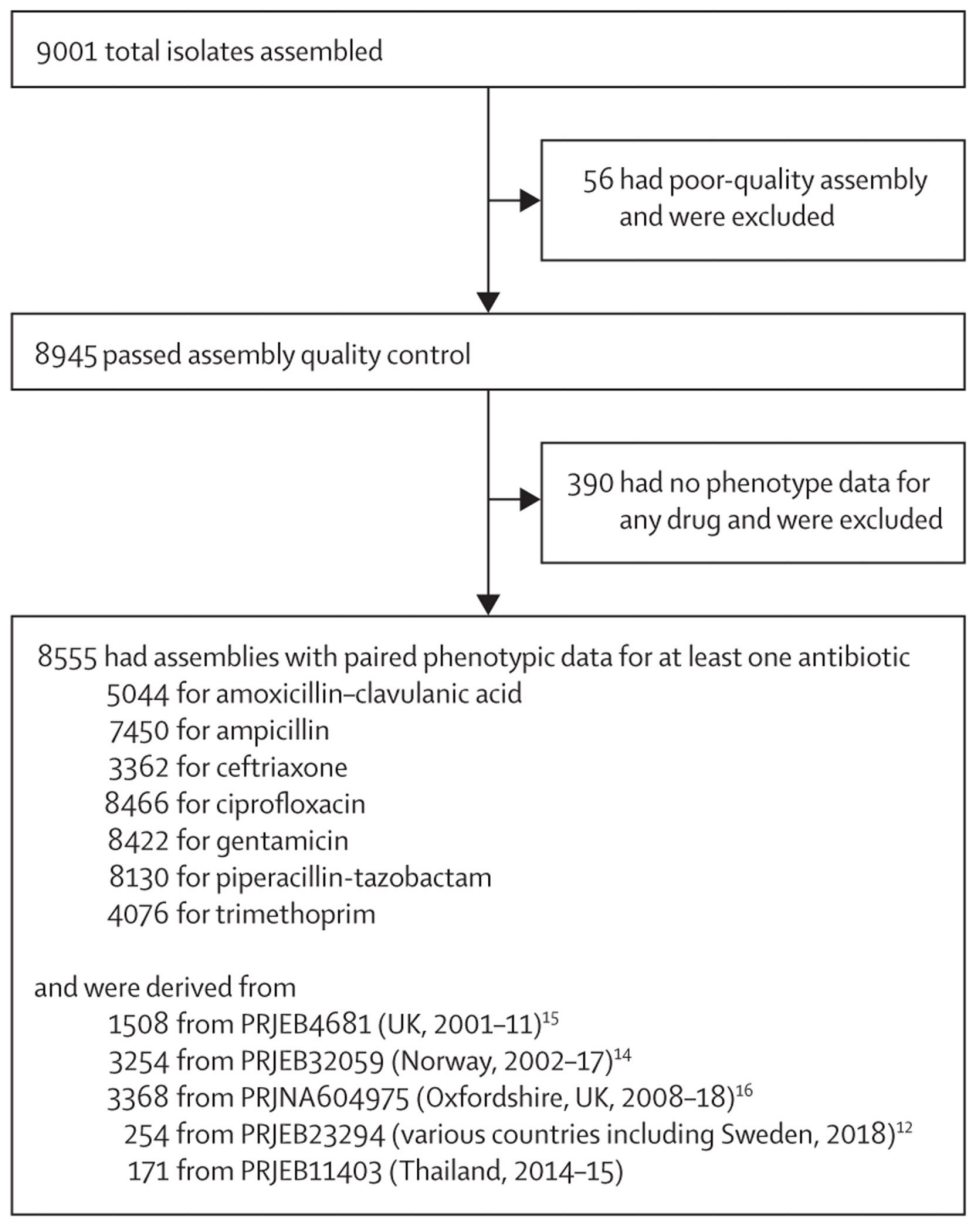
Isolates included and excluded in the analysis, and subsequent data availability for study components

We assembled a collection of 9001 *E coli* isolates, of which 8555 had linked whole-genome sequencing data and binary phenotypic classifications available for at least one antibiotic of interest (figure 1; appendix 1 p 3). We first investigated the proportion of AMR that could be explained using the current AMRFinder database. The sensitivity of the AMRFinder database (ie, percentage of phenotypically resistant isolates with a relevant ARG as determined by AMRFinder) using 100% identity or coverage filters was notably poorer for β-lactam–β-lactamase inhibitor combinations (24⋅6% [95% CI 22⋅5–26⋅8] for amoxicillin–clavulanic acid and 40⋅3% [35⋅0–45⋅9] for piperacillin–tazobactam) than other antibiotics considered, which had sensitivities in the range 86⋅7–91⋅8% (table 1, appendix 1 p 9). Conversely, specificity was high for all antibiotics (range 96⋅0–99⋅6%; 99⋅2% [98⋅9–99⋅5] for amoxicillin–clavulanic acid and 96⋅0% [95⋅5–96⋅4] for piperacillin–tazobactam). The sensitivity for both amoxicillin–clavulanic acid and piperacillin– tazobactam was improved by predicting isolates carrying *bla*_TEM-1_ as resistant, but this reduced specificity (increase in sensitivity 61⋅7% [61⋅1–62⋅0] *vs* 48⋅0% [45⋅6–49⋅2] but reduction in specificity 29⋅4% [28⋅2–30⋅7] *vs* 36⋅9% [36⋅2–37⋅5]; table 1).

Using 100% identity or coverage filters, no drugs met the FDA specified thresholds for very major error rates but six (amoxicillin–clavulanic acid, ampicillin, ceftriaxone, cipro-floxacin, gentamicin, and trimethoprim) met the thresholds for major error rates (table 1). Resampling simulations suggested that this finding is also likely to be applicable to settings with a higher prevalence of AMR (appendix 1 p 10). When identity or coverage settings were relaxed to default settings, there was an increase in sensitivity for six drugs (ampicillin, ciprofloxacin, ceftriaxone, gentamicin, and trimethoprim) but a drop in specificity for all except gentamicin. This change increased the major error rates for ciprofloxacin and ceftriaxone to above the FDA specified acceptable threshold (table 1). A sensitivity analysis revealed no improvement in performance when intermediate identity thresholds were trialled for ciprofloxacin (appendix 1 p 4). Results were broadly similar between the different datasets included (appendix 1 p 9), although there were some notable exceptions, including a higher major error rate for ciprofloxacin in the Thai collection of isolates (due to carriage of *gyrA* Ser83Leu in these isolates of 106 [66%] of 161 *vs* 1601 [19⋅3%] of 8305 in the rest of the dataset) and higher very major error rates for ampicillin (43⋅1% [95% CI 36⋅3–50⋅1]) and gentamicin (31⋅0% [19⋅9–44⋅7]) in the Swedish dataset, which was relatively enriched for antibiotic-resistant isolates (prevalence of ampicillin resistance 209 [82%] of 254 *vs* 1935 [57⋅1%] of 3390 in the Oxfordshire study, and 1279 [39⋅3%] of 3251 in the Norwegian study; prevalence of gentamicin resistance 58 [23%] of 254 *vs* 215 [6⋅3%] of 3397 in the Oxfordshire study and 152 [4⋅8%] of 3144 in the Norwegian study). We have compared the Oxfordshire and Norwegian studies directly because they are the two unselected longitudinal studies and, therefore, most epidemiologically representative.

**Figure 2:**
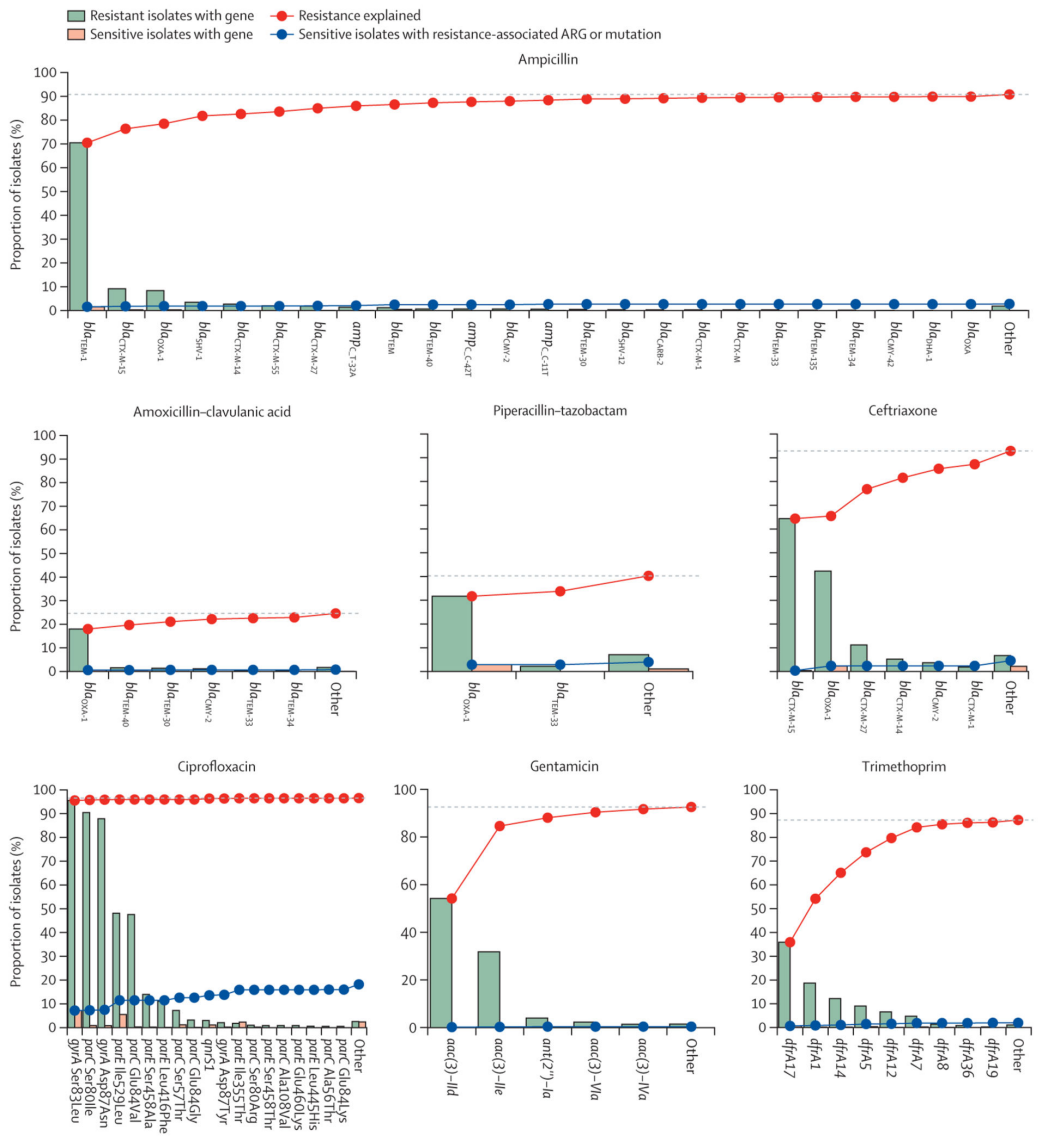
Proportion of all resistant isolates carrying known ARGs carrying known ARGs ARGs on the x-axis are ordered by their frequency in the dataset. Grey dashed lines show the maximum proportion of resistance potentially explainable by known ARGs. The sensitivity gap (1–sensitivity, the proportion of resistant isolates above this dashed line) varied by antibiotic: 9⋅2% for ampicillin, 75⋅4% for amoxicillin–clavulanic acid, 59⋅7% for piperacillin–tazobactam, 7⋅1% ceftriaxone, 3⋅4% ciprofloxacin, 7⋅4% gentamicin, and 12⋅7% trimethoprim. ARG=antibiotic resistance gene.

For all antibiotic classes, the majority of explainable resistance was conferred by a small number of ARGs or mutations in the AMRFinder database, with a large number of rarer alleles contributing relatively little (figure 2). The high major error rate for ciprofloxacin (table 1) was partly explained by the fact that although 1110 (95⋅6%) of 1161 resistant isolates had a *gyrA* Ser83Leu mutation, so did 528 (7⋅2%) of 7305 sensitive isolates. The greatest phenotypic variability occurred with carriage of *bla*_TEM-1_ for amoxicillin–clavulanic acid (1100 [52⋅0%] of 2115 resistant isolates) and piperacillin–tazobactam (194 [6⋅3%] of 3084 resistant isolates). All antibiotics had a sensitivity gap, a proportion of resistance that could not be explained using all ARGs or mutations included in the current AMRFinder catalogue (ie, 1–sensitivity shown in table 1 at the default identity thresholds), but this varied by drug, from 75⋅4% for amoxicillin–clavulanic acid to 3⋅4% for ciprofloxacin (figure 2).

There were 6682 (74⋅7%) of 8945 isolates with at least one AMRFinder hit among the antibiotic classes of interest. Only 18 199 (51⋅5%) of the 35 343 ARGs detected had a 100% amino acid identity and coverage match to the reference. From these 35 343 ARGs we detected 136 unique AMR-associated gene families containing 1042 unique alleles. 126 (12⋅1%) of these 1042 ARGs were present at least ten times in the dataset, of which 61 (48⋅4%) had a 100% amino acid identity to the reference sequence in the AMRFinder database; 313 (30⋅0%) of the 1042 ARGs were present between two and nine times, of which 110 (35⋅1%) had a 100% amino acid identity to the reference sequence. Alleles of *bla*_TEM-1_, *aph(6)-Id, aadA1, dfrA1, aph(3*”*)-Ib*, and *dfrA14* were among the most commonly observed uncatalogued ARGs (appendix 1 p 11). 603 (57⋅9%) of the 1042 unique ARGs were singletons (ie, occurred only once; appendix 1 p 12); these could either have a low phenotypic effect and therefore not be readily selected for, be associated with a high fitness cost and therefore be commonly lost, be currently rare (eg, because they have recently emerged), or be bioinformatic or sequencing noise.

There was no evidence of a difference in the proportion of singletons versus non-singletons that had a 100% amino acid match in the AMRFinder database (214 [35%] of 603 *vs* 171 [39%] of 439, p=0⋅28). We found similar average sequencing depths for singleton versus non-singleton genes (median 69 [IQR 50–100] *vs* 68 [46–106], p=0⋅47), suggesting that sequencing error is unlikely (appendix 1 p 13). Although assembly discrepancies between SKESA and Shovill assemblies (considering a random selection of 1000 isolates with at least one singleton ARG) were significantly more common in singleton versus non-singleton ARGs (11 [13%] of 87 *vs* 117 [1⋅7%] of 6762, p<0⋅0001), the fact that assemblies were consistent for the majority of singletons (76 [87%] of 87) suggests that the majority of these are still more likely to represent true background diversity rather than bioinformatic or sequencing noise.

**Figure 3:**
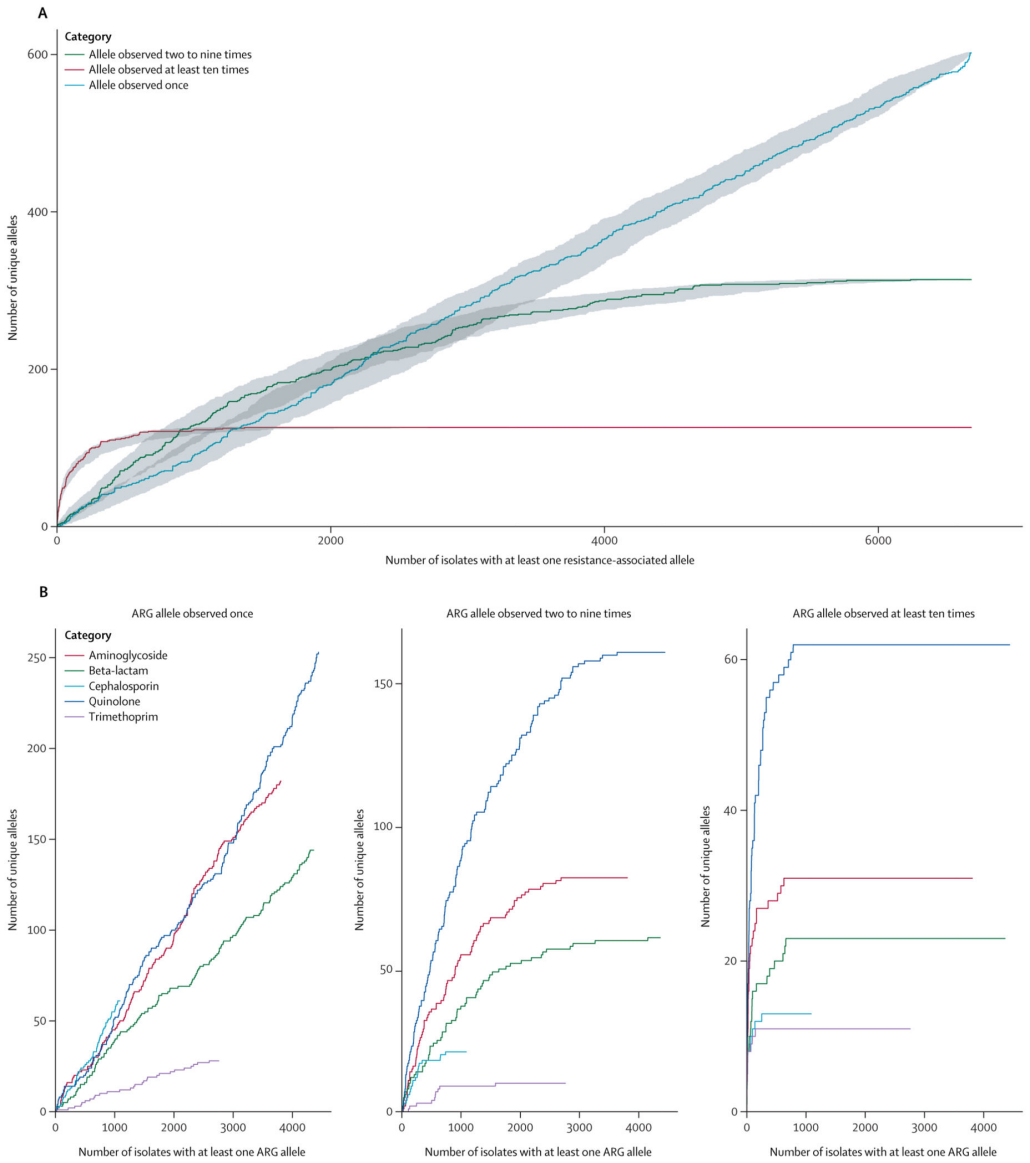
Simulated accumulation curves of total numbers of unique ARGs (A) Relationship between the total number of unique ARGs observed across all antibiotic classes and the number of isolates with at least one ARG in the dataset. Coloured lines represent median estimates with 95% CIs (estimated by bootstrap approximation) shown in grey. (B) Relationship between the total number of unique ARGs observed and the number of isolates with at least one ARG of the antibiotic class denoted by the colour of the line in the dataset. Note that the y-axis scale is different for each plot. ARG=antibiotic resistance gene.

Similar patterns of uncatalogued variation (ie, frequent singletons, fewer examples of gene variants that appear in two or more isolates, and the fewest examples appearing in ten or more isolates) in known ARGs were observed for all drugs and acrossstudies, with no evidence of a plateau in the rate of discovery of new singleton ARG alleles with increasing number of isolates (figure 3; appendix 1 pp 12, 14). By contrast, the accumulation curves for all drug classes plateaued when considering ARG alleles observed at least twice in the dataset, which might be less likely to be bioinformatic or sequencing noise.

Overall, there was a strong relationship between the number of times an ARG allele was observed in Oxfordshire isolates and the number of times it was observed in non-Oxfordshire isolates (Spearman coefficient 0⋅76 [95% CI 0⋅73–0⋅80], p<0⋅0001; appendix 1 p 15). For all drugs there were no ARG alleles observed six times or more in Oxfordshire isolates that were unique to this dataset (appendix 1 p 15).

Using the examples of the β-lactam–β-lactamase inhibitor combinations amoxicillin–clavulanic acid and piperacillin–tazobactam (which both currently have poorer genotype-to-phenotype predictive performance), we investigated whether uncatalogued variation in known AMR-associated gene families might have an important effect on AMR phenotype. There were 108 unique ARGs that clustered in the *bla*_TEM-1_ gene family, although the vast majority of sequences identified were one of four ARGs (here designated: the reference *bla*_TEM-1_1_, n=2469 [68⋅8%] of 3587; *bla*_TEM-1_2_, n=94 [2⋅6%]; *bla*_TEM-1_3_, n=561 [15⋅6%]; and *bla*_TEM-1_4_, n=225 [6⋅3%]). All four had identical amino acid sequences (and hence were indistinguishable to the AMRFinder tool), but there were six synonymous polymorphic sites distinguishing these alleles (appendix 1 p 16). The remaining 238 (6⋅6%) comprised 104 distinct ARGs, of which 53 (51⋅0%) were exact amino acid matches to known *bla*_TEM_ variants (eg, 27 *bla*_TEM-30_, 32 *bla*_TEM-40_, 12 *bla*_TEM-12_, and *bla*_TEM-33_). After adjusting for other known alleles predicted to confer amoxicillin–clavulanic acid resistance (by ResFinder), *bla*_TEM-1_2_ was associated with less resistance compared with the *bla*_TEM-1_1_ reference group (aOR 0⋅58 [95% CI 0⋅35–0⋅95], p=0⋅031; table 2). Similarly, *bla*_TEM-1_3_ was associated with reduced resistance to piperacillin–tazobactam (aOR 0⋅50 [0⋅29–0⋅82], p=0⋅0047). These associations remained significant after adjusting for population structure, although there was some evidence that this confounds the relationship between *bla*_TEM-1 3_ and reduced resistance to piperacillin–tazobactam (appendix 1 p 6).

## Discussion

We analysed the ARG content of a global collection of 9001 isolates (of which 8555 had phenotypic data) to investigate how much resistance to antibiotics commonly prescribed for *E coli* infections is explained by AMR-associated gene families included in existing catalogues, and the extent of uncatalogued variation with these. For all classes of antibiotics considered here, we found that the majority of resistance is conferred by a relatively small number of ARGs. However, the fact that the AMRFinder database did not meet FDA thresholds for any of the drugs evaluated in this study emphasises the need for identification of more AMR-associated gene families, and better refinement of genotype–phenotype correlations. We showed that there is substantial background variation within known AMR-associated gene families, and that better cataloguing of this (including synonymous mutations) could improve phenotypic predictions. Although most uncatalogued alleles were rare, those that occurred at least six times in the Oxfordshire dataset were always also observed elsewhere, indicating strong selective pressures for convergent evolution or rapid global dissemination of successful genomic variation.

For all drugs considered, there was a proportion of resistance (around 10% for most drugs, higher for β-lactam–β-lactamase inhibitor combinations, and slightly lower for ciprofloxacin) that could not be explained by the presence or absence of known AMR-associated gene families. We hypothesise that this sensitivity gap partly comprises laboratory mistakes, mislabelling, or technical failure; partly of AMR-associated gene families yet to be discovered; and partly because of phenotypic resistance conferred by, for example, promoter mutations, combinations of genes, and differential copy number or expression that are currently not effectively captured in current genotypic catalogues. The fact that the most known resistance is conferred by relatively few alleles suggests that very large datasets will be needed to power studies to discover new AMR-associated gene families. An alternative approach using in vitro mutagenesis or synthetic biology could complement and speed up discovery, and enable a more refined understanding of the specific effects of mutations on resistance phenotype and fitness.^26^

Most existing epidemiology and resistance prediction studies report ARG presence or absence using default thresholds and do not further categorise uncatalogued variation within gene families. This binary presence or absence approach is in contrast to the efforts made to catalogue the phenotypic effects of every mutation within AMR-associated gene families in *Mycobacterium tuberculosis*,^27^ although, notably, the number of gene targets is much smaller than for *E coli*. The tacit assumption of a binary presence or absence approach is that the cloud of genetic variation observed in known resistance-encoding targets is either biologically unimportant or represents an artefact created by sequencing or bioinformatic errors, and does not have an important effect on phenotype; we have shown here that this is not the case. The frequent presence of uncharacterised genetic variation with an unknown effect on phenotype is problematic for potential clinical application of existing databases. We hypothesise that analysis of minimum inhibitory concentration data would further illustrate this point by providing evidence of small but potentially important incremental effects of variation on resistance. Our data also show that new ARG variants are being continuously generated, highlighting the potential risk that this kind of rapid genetic churn might quickly generate extended resistance phenotypes, as has been shown with *bla*_KPC_ and resistance to ceftazidime-avibactam.^28^

Although our study was not primarily designed to assess the AMRFinder tool, by evaluating its performance on a dataset of 8586 isolates, we have nevertheless conducted the largest such validation to date. None of the antibiotic classes evaluated met the FDA criteria for acceptable major error and very major error rates. For ciprofloxacin, several common resistance-associated mutations did not invariably cause resistance when present in isolation, resulting in a high major error rate (particularly for isolates from Thailand) that could cause unnecessary avoidance of this antibiotic were AMRFinder to be used without further expert interpretation in a clinical setting. More granular drug-level classification of ARGs should be a priority for the AMRFinder tool and would likely improve predictive performance (as exemplified by the overall slightly better performance of ResFinder in a recent validation study,^10^ and by better performance of existing tools when using curated gene–drug associations).^9,29^ Our study also highlights the potential phenotypic importance of synonymous mutations, suggesting that classification of ARGs using amino acid sequences alone should be avoided.

Limitations of this study include the fact that the dataset over-represents European bloodstream infection isolates (8130 [95⋅0%] of 8555). There is a risk of bias from over-representing clonal isolates in outbreaks, which was minimised by the inclusion of large unselected longitudinal studies. Although our data suggest that novel variants of known ARGs are selected across geographical contexts, and that the AMRFinder tool performs similarly in the studies included in this analysis, there is a risk of extrapolating performance characteristics from evaluations on northern European datasets to areas with a higher incidence of AMR. More data to evaluate the emergence and selection of novel ARG alleles in higher-incidence settings will be valuable to evaluate optimal sampling strategies for ongoing surveillance efforts. Although all included studies used European Committee on Antimicrobial Susceptibility Testing break-points, it is possible that differences in standard operating procedures and use of different versions of the Committee guidelines might explain some of the variation of phenotypes and concordance with genotype. Resistance to β-lactam–β-lactamase inhibitor (and possibly other) antibiotics might be explained by considering other factors related to ARG presence that we did not explore here.^11^ For some classes of antibiotics (eg, fluoroquinolones),^30^ resistance-associated mutations or ARGs are found in phenotypically susceptible isolates; more complex models accounting for this might perform better than the simple presence or absence interpretation of genotype evaluated in this study. The statistical associations identified in this study between variants of *bla*_TEM-1 1_ and reduced or increased relative susceptibility to β-lactam–β-lactamase inhibitor combinations require further experimental validation. Although we found no evidence of a difference in ARG content in excluded isolates, the missing antibiotic resistance phenotype data for 390 (4⋅4%) of 8945 isolates are an additional limitation, as is the fact that we only evaluated a single ARG database.

In summary, we have shown substantial variation in known AMR gene targets in *E coli*, some of which are selected across space and time. Surveillance approaches taking this uncatalogued variation into account might be able to more rapidly identify genetic variants that are emerging or disseminating. We highlight three areas of focus for the improvement of existing ARG databases. For most drug classes, current knowledge explains most, but not all, resistance and so new gene target discovery is needed. For new, as yet undiscovered AMR-associated gene families, it will be important to develop rules for systematically cataloguing new alleles so that their phenotypic effect can be properly considered. Finally, the application of databases needs to be improved to consider mutations at both the nucleotide and amino acid level, as well as the effects of these changes on phenotypes at the specific drug species level.

## Supplementary Material

Supplementary appendix

Supplementary dataset

## Figures and Tables

**Table 1:** Performance metrics for the ability of the AMRFinder database to predict phenotype from genotype

	Concordance	Sensitivity	Specificity	NPV	PPV	Major error	Very major error
**Amoxicillin–clavulanic acid, 1607 (31⋅9%) of 5044 isolates phenotypically resistant** †							
Default coverage or ID threshold	75⋅5% (74⋅2–76⋅6)	24⋅6% (22⋅5–26⋅8)	99⋅2% (98⋅9–99⋅5)	73⋅8% (72⋅5–75⋅0)	93⋅8% (91⋅0–95⋅8)	0⋅8% (0⋅5–1⋅1)	75⋅4% (73⋅2–77⋅5)*
100/100 coverage or ID threshold	75⋅5% (74⋅2–76⋅6)	24⋅6% (22⋅5–26⋅8)	99⋅2% (98⋅9–99⋅5)	73⋅8% (72⋅5–75⋅0)	93⋅8% (91⋅0–95⋅8)	0⋅8% (0⋅5–1⋅1)	75⋅4% (73⋅2–77⋅5)*
**Amoxicillin–clavulanic acid plus *bla*_TEM-1_, 1607 (31⋅9%) of 5044 isolates phenotypically resistant** †							
Default coverage or ID threshold	74⋅8% (73⋅6–76⋅0)	87⋅5% (85⋅8–89⋅1)	68⋅9% (67⋅3–70⋅5)	92⋅2% (91⋅1–93⋅2)	56⋅8% (54⋅8–58⋅8)	31⋅1% (29⋅5–32⋅7)*	12⋅5% (10⋅9–14⋅2)*
100/100 coverage or ID threshold	75⋅1% (73⋅8–76⋅2)	86⋅3% (84⋅5–87⋅9)	69⋅8% (68⋅2–71⋅3)	91⋅6% (90⋅5–92⋅6)	57⋅2% (55⋅2–59⋅2)	30⋅2% (28⋅7–31⋅8)*	13⋅7% (12⋅1–15⋅5)*
**Ampicillin, 3821 (51⋅3%) of 7450 isolates phenotypically resistant**							
Default coverage or ID threshold	93⋅9% (93⋅3–94⋅4)	90⋅8% (89⋅8–91⋅7)	97⋅2% (96⋅6–97⋅7)	90⋅9% (90⋅0–91⋅8)	97⋅1% (96⋅5–97⋅6)	2⋅8% (2⋅3–3⋅4)	9⋅2% (8⋅3–10⋅2)*
100/100 coverage or ID threshold	92⋅9% (92⋅3–93⋅5)	88⋅1% (87⋅1–89⋅2)	97⋅9% (97⋅3–98⋅3)	88⋅7% (87⋅7–89⋅6)	97⋅8% (97⋅2–98⋅2)	2⋅1% (1⋅7–2⋅7)	11⋅9% (10⋅9–12⋅9)*
**Ceftriaxone, 267 (7⋅9%) of 3362 isolates phenotypically resistant**							
Default coverage or ID threshold	95⋅2% (94⋅4–95⋅9)	92⋅9% (88⋅9–95⋅5)	95⋅4% (94⋅6–96⋅1)	99⋅4% (99⋅0–99⋅6)	63⋅4% (58⋅4–68⋅2)	4⋅6% (3⋅9–5⋅4)*	7⋅1% (4⋅5–11⋅1)*
100/100 coverage or ID threshold	96⋅9% (96⋅2–97⋅5)	91⋅0% (86⋅8–94⋅0)	97⋅4% (96⋅8–97⋅9)	99⋅2% (98⋅8–99⋅5)	75⋅2% (70⋅0–79⋅8)	2⋅6% (2⋅1–3⋅2)	9⋅0% (6⋅0–13⋅2)*
**Ciprofloxacin, 1161 (13⋅7%) of 8466 isolates phenotypically resistant**							
Default coverage or ID threshold	83⋅8% (83⋅0–84⋅6)	96⋅6% (95⋅4–97⋅6)	81⋅8% (80⋅9–82⋅7)	99⋅4% (99⋅1–99⋅5)	45⋅8% (43⋅8–47⋅8)	18⋅2% (17⋅3–19⋅1)*	3⋅4% (2⋅4–4⋅6)
100/100 coverage or ID threshold	85⋅6% (84⋅9–86⋅4)	3⋅9% (2⋅9–5⋅2)	98⋅6% (98⋅3–98⋅9)	86⋅6% (85⋅8–87⋅3)	31⋅0% (23⋅8–39⋅3)	1⋅4% (1⋅1–1⋅7)	96⋅1% (94⋅8–97⋅1)*
**Gentamicin, 648 (7⋅7%) of 8422 isolates phenotypically resistant**							
Default coverage or ID threshold	99⋅0% (98⋅8–99⋅2)	92⋅6% (90⋅2–94⋅4)	99⋅6% (99⋅4–99⋅7)	99⋅4% (99⋅2–99⋅5)	94⋅6% (92⋅5–96⋅2)	0⋅4% (0⋅3–0⋅6)	7⋅4% (5⋅5–9⋅8)*
100/100 coverage or ID threshold	99⋅0% (98⋅8–99⋅2)	91⋅8% (89⋅4–93⋅8%)	99⋅6% (99⋅5–99⋅7)	99⋅3% (99⋅1–99⋅5)	95⋅4% (93⋅3–96⋅8)	0⋅4% (0⋅3–0⋅5)	8⋅2% (6⋅2–10⋅6)*
**Piperacillin–tazobactam, 325 (4⋅0%) of 8130 isolates phenotypically resistant**							
Default coverage or ID threshold	93⋅8% (93⋅2–94⋅3)	40⋅3% (35⋅0–45⋅9)	96⋅0% (95⋅5–96⋅4)	97⋅5% (97⋅1–97⋅8)	29⋅6% (25⋅4–34⋅1)	4⋅0% (3⋅6–4⋅5)*	59⋅7% (54⋅1–65⋅0)*
100/100 coverage or ID threshold	93⋅8% (93⋅2–94⋅3)	40⋅3% (35⋅0–45⋅9)	96⋅0% (95⋅5–96⋅4)	97⋅5% (97⋅1–97⋅8)	29⋅6% (25⋅4–34⋅1)	4⋅0% (3⋅6–4⋅5)*	59⋅7% (54⋅1–65⋅0)*
**Piperacillin–tazobactam plus *bla*_TEM-1_, 325 (4⋅0%) of 8130 isolates phenotypically resistant** †							
Default coverage or ID threshold	60⋅3% (59⋅2–61⋅3)	88⋅3% (84⋅2–91⋅5)	59⋅1% (58⋅0–60⋅2)	99⋅2% (98⋅9–99⋅4)	8⋅2% (7⋅4–9⋅2)	40⋅9% (39⋅8–42⋅0)*	11⋅7% (8⋅5–15⋅8)*
100/100 coverage or ID threshold	60⋅3% (59⋅2–61⋅3)	88⋅3% (84⋅2–91⋅5)	59⋅1% (58⋅0–60⋅2)	99⋅2% (98⋅8–99⋅4)	8⋅3% (7⋅5–9⋅3)	40⋅9% (39⋅8–42⋅0)*	11⋅7% (8⋅5–15⋅8)*
**Trimethoprim, 1506 (36⋅9%) of 4076 isolates phenotypically resistant**							
Default coverage or ID threshold	94⋅0% (93⋅3–94⋅7)	87⋅3% (85⋅5–88⋅9)	97⋅3% (97⋅3–98⋅5)	92⋅9% (91⋅9–93⋅9)	96⋅2% (95⋅0–97⋅1)	2⋅0% (1⋅5–2⋅7)	12⋅7% (11⋅1–14⋅5)*
100/100 coverage or ID threshold	94⋅0% (93⋅2–94⋅7)	86⋅7% (84⋅9–88⋅4)	98⋅2% (97⋅6–98⋅7)	92⋅7% (91⋅6–93⋅6)	96⋅6% (95⋅4–97⋅5)	1⋅8% (1⋅3–2⋅4)	13⋅3 (11⋅6–15⋅1)*

Data are % (95% CI). Coverage or ID threshold refers to the percentage of amino acid coverage or identity of the gene identified by AMRFinder compared with the reference. ID=identity. NPV=negative predictive value. PPV=positive predictive value.

* Performance metrics that did not meetthe US Food and Drug Administration thresholds for major discrepancies (major error<3%) or very major discrepancies (very major errorupperbound of 95% CI <7⋅5%).

† *bla*_TEM-1_ is classified as conferring resistance against amoxicillin–clavulanic acid or piperacillin–tazobactam.

**Table 2:** Univariable and multivariable associations of ARGs shown with amoxicillin–clavulanic acid and piperacillin–tazobactam resistance

	Univariable regression analysis for amoxicillin–clavulanic acid			Multivariable regression analysis for amoxicillin–clavulanic acid			Univariable regression analysis for piperacillin–tazobactam			Multivariable regression analysis for piperacillin–tazobactam	
	OR	p		aOR	p		OR	p		aOR	p
*bla*_TEM-1_1_	1⋅00 (reference)	⋅⋅		1⋅00 (reference)	⋅⋅		1⋅00 (reference group)	⋅⋅		1⋅00 (reference group)	⋅⋅
*bla*_TEM-1_2_	0⋅51 (0⋅31–0⋅83)	0⋅0068		0⋅58 (0⋅35–0⋅95)	0⋅031		0⋅69 (0⋅23–1⋅62)	0⋅43		0⋅87 (0⋅28–2⋅04)	0⋅76
*bla*_TEM-1_3_	0⋅89 (0⋅70–1⋅11)	0⋅30		1⋅00 (0⋅79–1⋅26)	0⋅98		0⋅42 (0⋅24–0⋅68)	<0⋅0001		0⋅50 (0⋅29–0⋅82)	0⋅0047
*bla*_TEM-1_4_	1⋅19 (0⋅85–1⋅67)	0⋅31		1⋅34 (0⋅96–1⋅89)	0⋅085		0⋅88 (0⋅47–1⋅51)	0⋅66		1⋅10 (0⋅59–1⋅90)	0⋅74
*bla*_TEM-1_other_	2⋅13 (1⋅52–3⋅04)	<0⋅0001		2⋅29 (1⋅62–3⋅28)	<0⋅0001		1⋅73 (1⋅09–2⋅66)	0⋅021		1⋅98 (1⋅23–3⋅07)	0⋅0057
*bla*_CMY-2_	23⋅7 (3⋅17–3035⋅88)	<0⋅0001		21⋅38 (2⋅79–2745⋅94)	<0⋅0001		2⋅43 (0⋅48–7⋅98)	0⋅25		1⋅90 (0⋅35–6⋅65)	0⋅41
*bla*_OXA-1_	23⋅05 (9⋅13–83⋅21)	<0⋅0001		24⋅52 (9⋅69–88⋅65)	<0⋅0001		8⋅80 (5⋅55–13⋅75)	<0⋅0001		8⋅68 (5⋅41–13⋅72)	<0⋅0001
*bla*__other_	29⋅06 (3⋅93–3707⋅68)	<0⋅0001		31⋅22 (4⋅21–3986⋅66)	<0⋅0001		14⋅52 (5⋅46–38⋅62)	<0⋅0001		13⋅46 (4⋅89–36⋅67)	<0⋅0001

Data are OR (95% CI), aOR (95% CI), or p value. *bla*_TEM-1_1_, *bla*_TEM-1_2_, *bla*_TEM-1_3_,and *bla*_TEM-1_4_ denote the four most common alleles of *bla*_TEM-1_ in the dataset (all others are denoted as *bla*_TEM-1_other_). *bla*_TEM-1_1_ is the reference version of the gene (ie, 100% nucleotide match to the version found in the AMRFinder database). In the multivariable models, estimates are adjusted for the independent presence of the two most common β-lactam–β-lactamase inhibitor resistance-conferring ARGs (*bla*_CMY-2_, *bla*_OXA-1_, and of any other β-lactam–β-lactamase inhibitor ARGs grouped as *bla*_other_). aOR=adjusted odds ratio. ARG=antibiotic-resistance gene. OR=odds ratio.

## Data Availability

All assemblies are available at https://doi.org/10.6084/m9.figshare.22220212.v1 and associated metadata can be found in appendix 2. A file containing extracted ARG alleles can be downloaded from https://doi.org/10.6084/m9.figshare.25243165.v1. All code used for the analysis can be found at https://github.com/samlipworth/resistome_variation where there is also a binder environment in which the key aspects of the analysis can be replicated.

## Research in context

### Evidence before this study

We searched PubMed from database inception to March 16, 2023, using the terms *Escherichia coli* AND (antimicrobial resistance gene) AND ((catalogue) OR (database)) AND ((variation) OR (uncatalogued)). The 16 studies identified were mostly molecular epidemiology studies describing the distribution of antibiotic resistance genes (ARGs; identified using standard, non-exact identity and coverage thresholds) in various countries and reservoirs. None of the studies considered uncatalogued variation in ARGs.

### Added value of this study

To our knowledge, this is the first study to systematically examine uncatalogued variation within known resistance genes in *E coli*. Using a large global dataset of clinical isolates, we show that such variation is common, and that in the case of *bla*_TEM-1_ synonymous variation is associated with substantial phenotypic differences compared with the reference. Our analysis suggests that there is a constant background of genetic variation that undergoes strong selection pressures, emphasising the importance of stewardship to avoid the emergence and dissemination of variants with extended resistance phenotypes. We also show that, for all drugs, there is a proportion of resistance that cannot be explained by current resistance gene families, and that the widely used AMRFinder database does not meet US Food and Drug Administration specified performance metrics for predicting phenotype for most classes of antibiotics.

### Implications of all the available evidence

We identify two priorities for the improvement of existing ARG catalogues for *E coli*: identification of new resistance gene families associated with AMR, and improved cataloguing of genetic variation within known ones. We show that variation within known resistance genes (including synonymous mutations that can only be detected by analysing nucleotide sequences) can have substantial phenotype-modifying effects, which should prompt more detailed consideration in future epidemiology and resistance prediction studies. ARG detection based on amino acid sequence analysis alone should be avoided.
