# Supplementary material for: Exploring uncatalogued genetic variation in antimicrobial resistance gene families in Escherichia coli: an observational analysis
Source: Lancet Microbe. Author manuscript; Available in PMC 2025 Mar 10. (PMC7617469; doi:10.1016/S2666-5247(24)00152-6)
Supplement: Supplementary appendix [file EMS203364-supplement-Supplementary_appendix.pdf]

THE LANCET
Microbe

Supplementary appendix 1

This appendix formed part of the original submission and has been peer reviewed. We post it as supplied by the authors.

Supplement to: Lipworth S, Crook D, Walker AS, et al. Exploring uncatalogued genetic variation in antimicrobial resistance gene families in *Escherichia coli*: an observational analysis. *Lancet Microbe* 2024. https://doi.org/10.1016/S2666-5247(24)00152-6

Exploring uncatalogued genetic variation in antimicrobial resistance gene families in Escherichia coli

Supplementary Material

Contents

2 - Supplementary Methods

3 - Table S1

4 - Table S2

6 - Table S3

8 - Figure S1

9 - Figure S2

10 - Figure S3

11 - Figure S4

12 - Figure S5

13 - Figure S6

14 - Figure S7

15 - Figure S8

16 - Figure S9

17 - Supplementary references

Supplementary Methods

Checks for possible sequencing/bioinformatic error

We firstly evaluated the median read depth in singleton vs non-singleton ARGs. For each isolate, we used Snippy (v4.6.0)[25] to map raw reads back to the FASTA file created by the AMRFinder --nucleotide-output option. We then used samtools depth -a to evaluate the depth at every position in all ARGs and then summarised this output to give a median depth for each ARG. A Wilcoxon Rank Sum test was used to compare the distributions of read depths in singleton vs non-singleton ARGs. The assumption was that variants generated through sequencing error may occur due to lower read depth. We also selected 1000 random isolates (sort -R all isolates | tail -1000) with at least 1 singleton ARG and reassembled these using SKESA[26] (v.2.4.0) to evaluate the proportion of singleton ARGs that were identically assembled using two independent assembly methods (i.e. Shovill and SKESA).

Evaluation of ARG carriage in isolates included in/excluded from the study

We conducted a permanova (R package vegan[1]) to compare jaccard distances (vegdist, calculated from an ARG presence/absence matrix) between included and excluded isolates with 999 permutations. There was no evidence of a difference in ARG content between included and excluded isolates (R^2 = 0.00026, p=0.10).

Adjustment for population structure in supplementary table 3

Assemblies were sketched using Mash[2] (v2.3) with default settings (-s 1000, -k 21) and an all by all distance matrix subsequently created using Mash dist. A multidimensional scaling with five dimensions was created from this using cmdscale[3] in R. These five dimensions were then included as terms in the multivariable firth regression models.

Re-sampling simulations for Figure S3

We randomly selected isolates (with replacement) with weighting of resistant isolants to simulate prevalences from 5-50% in steps of 5. Test statistics (e.g. concordance, negative and positive predictive value, sensitivity and specificity and major and very major error) were calculated as before and resistance was predicted in the same manner as for table 1. For each prevalence threshold, this procedure was repeated 100 times. We then calculated medians from these simulated distributions.

Table S1 - details of phenotyping methods used in studies from which isolates included in this study were taken from

Gladstone et al[4]	Antimicrobial susceptibility data were generated by different methods - (i) Etest (2002-2005), (ii) disc diffusion using semi confluent growth (2006- 2010) and (iii) the European Committee on Antimicrobial Susceptibility Testing (EUCAST) disc diffusion method (2011-2017). Categorical interpretation (S/I/R) was according to the breakpoints for that specific year.
Kallonen et al[5]	Antimicrobial susceptibility testing was performed using the Vitek2 instrument with the N206 card (bioMerieux) for isolates from the CUH and using the agar dilution method for the BSAC collection.
Runcharoen et al[6]	Antimicrobial susceptibility testing was repeated using the N206 card on the Vitek 2 instrument (bioMérieux, Marcy l'Étoile, France) calibrated against EUCAST breakpoints, and these results were used during the analysis.
Lipworth et al[7]	Prior to 2013, antimicrobial susceptibility testing was performed using disk diffusion; after this, the Phoenix BD system was used with European Committee on Antimicrobial Susceptibility Testing (EUCAST) breakpoints.
Moradigaravand et al[8]	"Concentrations used were determined by performing a 2-fold serial dilution, starting from twice the concentrations listed by the European Committee on Antimicrobial Susceptibility Testing (EUCAST) on 25/01/2017, until no growth was observed after 16 hours for the common lab strain BW25113 used as a control in the experiments"

Table S2 - sensitivity analysis to investigate the effect of changing the % identity threshold filter in AMRFinder on the sensitivity/specificity for predicting Ciprofloxacin resistance.

% identity threshold	Sensitivity	Specificity
90	96.6	81.8
91	96.6	81.8
92	96.6	81.8
93	96.6	81.8
94	96.6	81.8
95	96.6	81.8

96	96.6	81.8
97	96.6	81.8
98	96.6	81.8
99	94.4	86.4
100	4.0	98.6

Table S3 - comparison of multivariable associations of blaTEM-1 alleles with co-amoxiclav and piperacillin-tazobactam resistance with and without adjustment for population structure.

**Drug/Variable**	**OR (95% CI) for multivariable model with no adjustment for population structure**	**p value for multivariable model with no adjustment for population structure**	**OR (95% CI) for multivariable model adjusted for population structure**	**p value for multivariable model adjusted for population structure**
**Co-amoxiclav**				
blaTEM-1_1	-	-	-	-
blaTEM-1_2	0.58 (0.35-0.95)	0.03	0.50 (0.30-0.83)	0.008
blaTEM-1_3	1.00 (0.79-1.26)	0.98	1.17 (0.91-1.50)	0.22
blaTEM-1_4	1.34 (0.96-1.89)	0.08	1.28 (0.90-1.82)	0.16
blaTEM-1_other	2.29 (1.62-3.28)	<0.001	2.20 (1.55-3.16)	<0.001
blaCMY-2	21.38 (2.79-2745.94)	<0.001	23.12 (2.99-2974.36)	<0.001
blaOXA-1	24.52 (9.59-88.65)	<0.001	20.87 (8.18-75.79)	<0.001
Other gene	31.22 (4.21-3986.66)	<0.001	35.51 (4.72-4545.84)	<0.001
**Piperacillin-tazobactam**				
blaTEM-1_1	-	-	-	-
blaTEM-1_2	0.87 (0.28-2.04)	0.76	1.03 (0.33-2.52)	0.95
blaTEM-1_3	0.50 (0.29-0.82)	0.005	0.57 (0.31-0.996)	0.048
blaTEM-1_4	1.10 (0.59-1.90)	0.74	0.82 (0.37-1.61)	0.59

blaTEM-1_other	1.98 (1.23-3.07)	0.006	2.00 (1.20-3.21)	0.008
blaCMY-2	1.90 (0.35-6.65)	0.41	1.87 (0.31-7.37)	0.45
blaOXA-1	8.68 (5.41-13.72)	<0.001	7.88 (4.60-13.38)	<0.001
Other gene	13.46 (4.89-36.67)	<0.001	8.43 (2.98-23.36)	<0.001

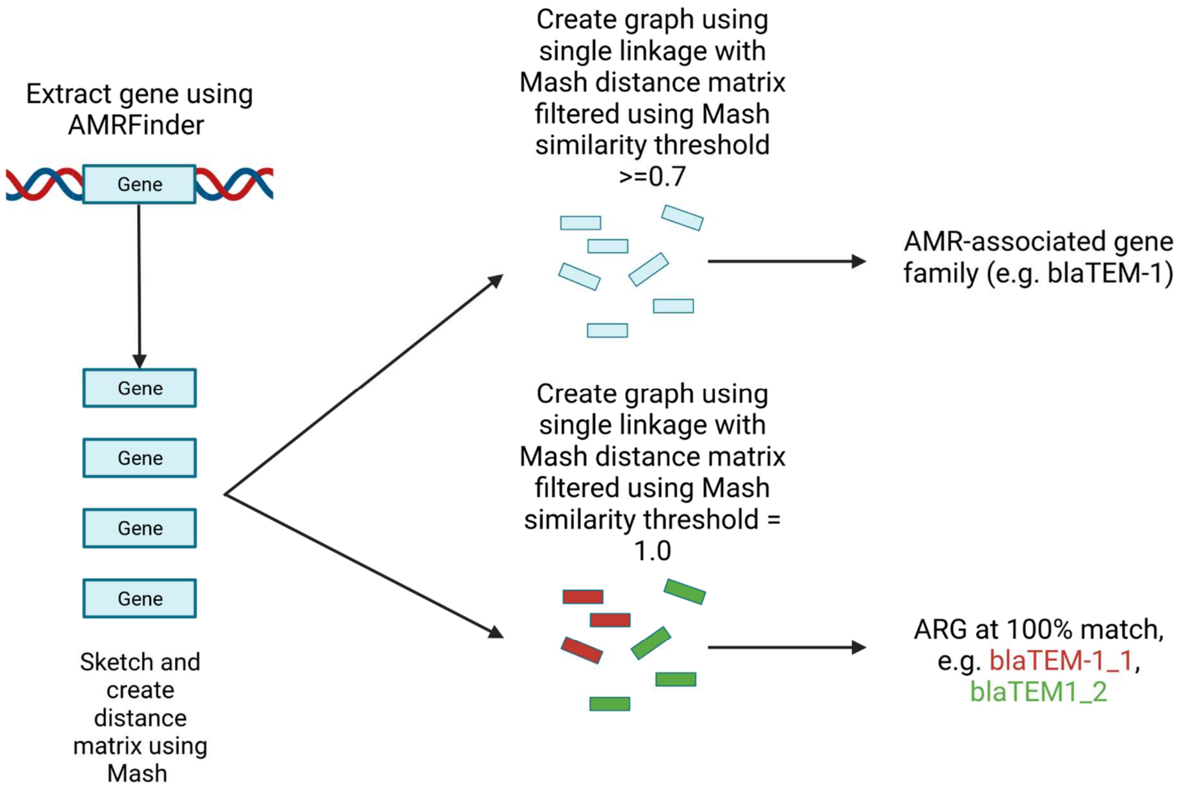

Figure S1 - Method used for empirical definition of AMR-associated gene families (clusters of genes identified at 70% similarity threshold) and ARGs (clusters of genes identified at 100% similarity threshold).

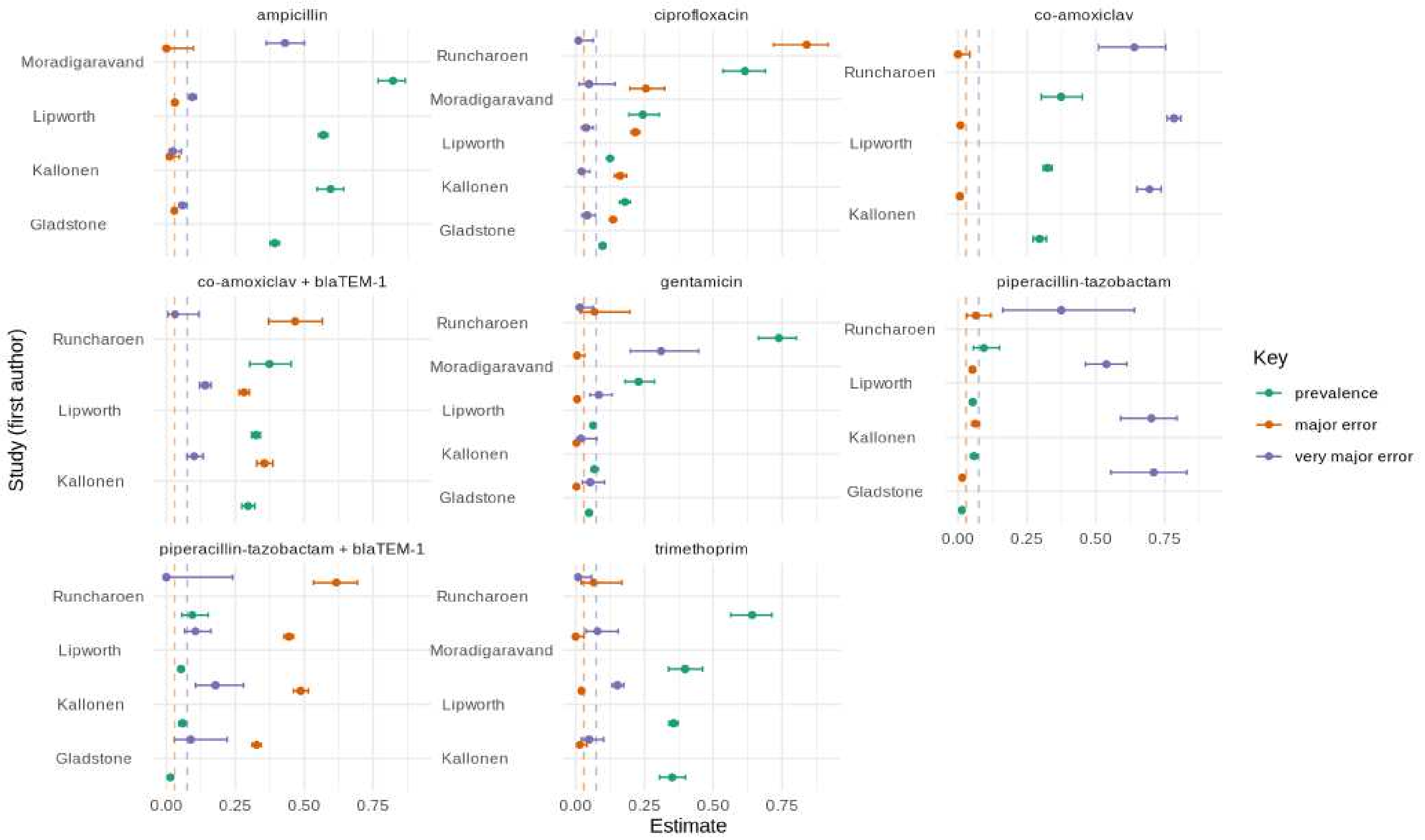

Figure S2 - performance of the AMRFinder database broken down by study. Circles show point estimates for the statistics shown in the key and error bars show 95% confidence intervals of these. Co-amoxiclav/piperacillin-tazobactam + blaTEM-1 refers to the fact that in these evaluations, blaTEM-1 was considered to confer resistance to the respective antibiotic. Hashed vertical lines show the position of the FDA thresholds for major error (<3%, orange) and very major error (upper bound of 95% confidence interval <7.5%,

purple). Corresponding references in the main text/project accessions for studies in this figure - Moradigaravand (Various including Sweden -12), Lipworth (Oxfordshire, UK - 16), Kallonen (UK - 15), Gladstone (Norway - 14), Runcharoen (Thailand - PRJEB11403).

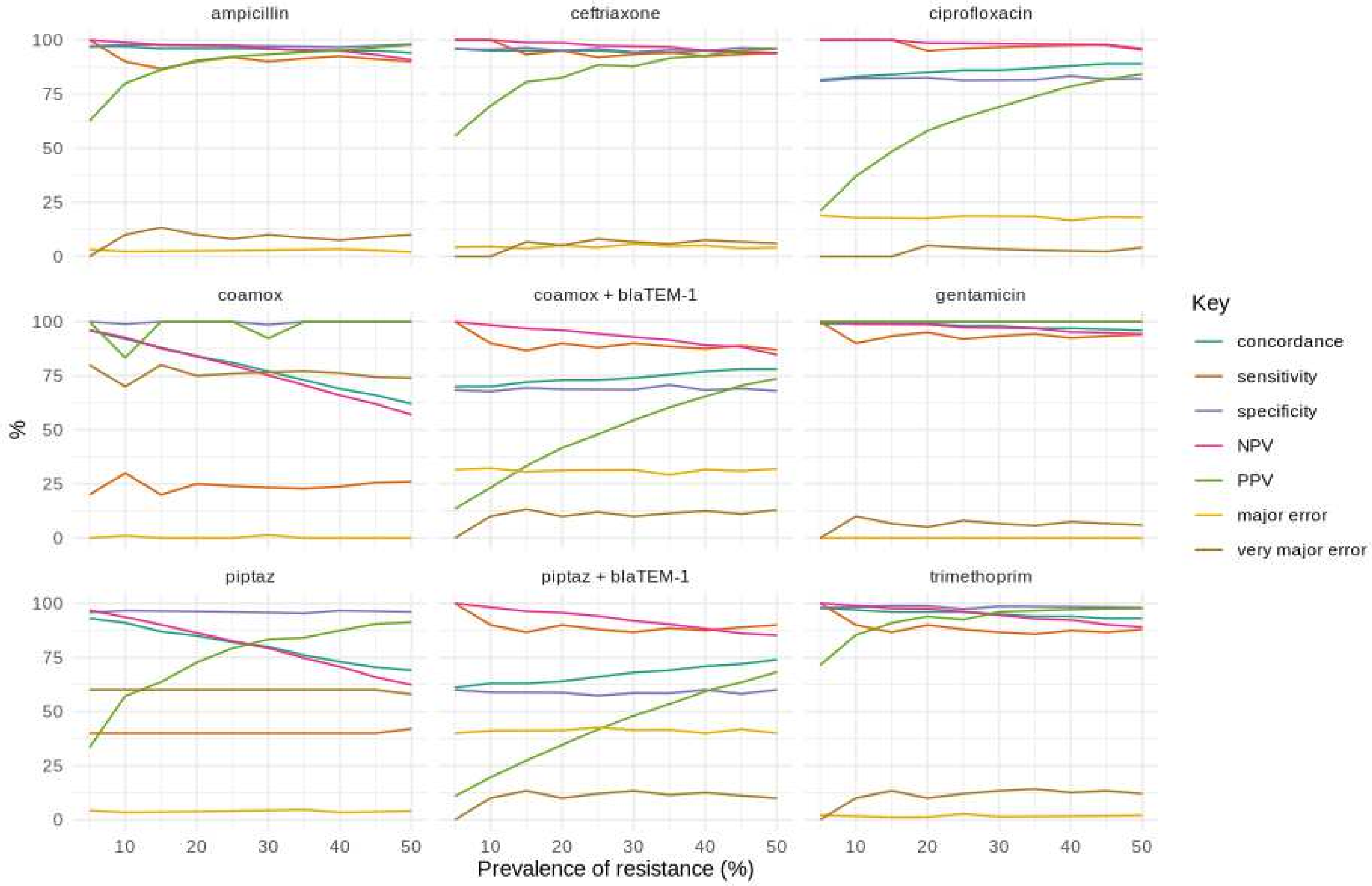

Figure S3 - re-sampling simulations to investigate the effect of AMR prevalence on the test statistics used to evaluate the AMRFinder database. The test statistics shown were recalculated for each of 100 random samples weighted to simulate the prevalence shown on the X-axis and median values subsequently plotted. Coamox/piptaz + bla_{TEM-1} refer to the evaluation conducted whereby isolates carrying bla_{TEM-1} were predicted to be resistant to co-amoxiclav/piperacillin-tazobactam.

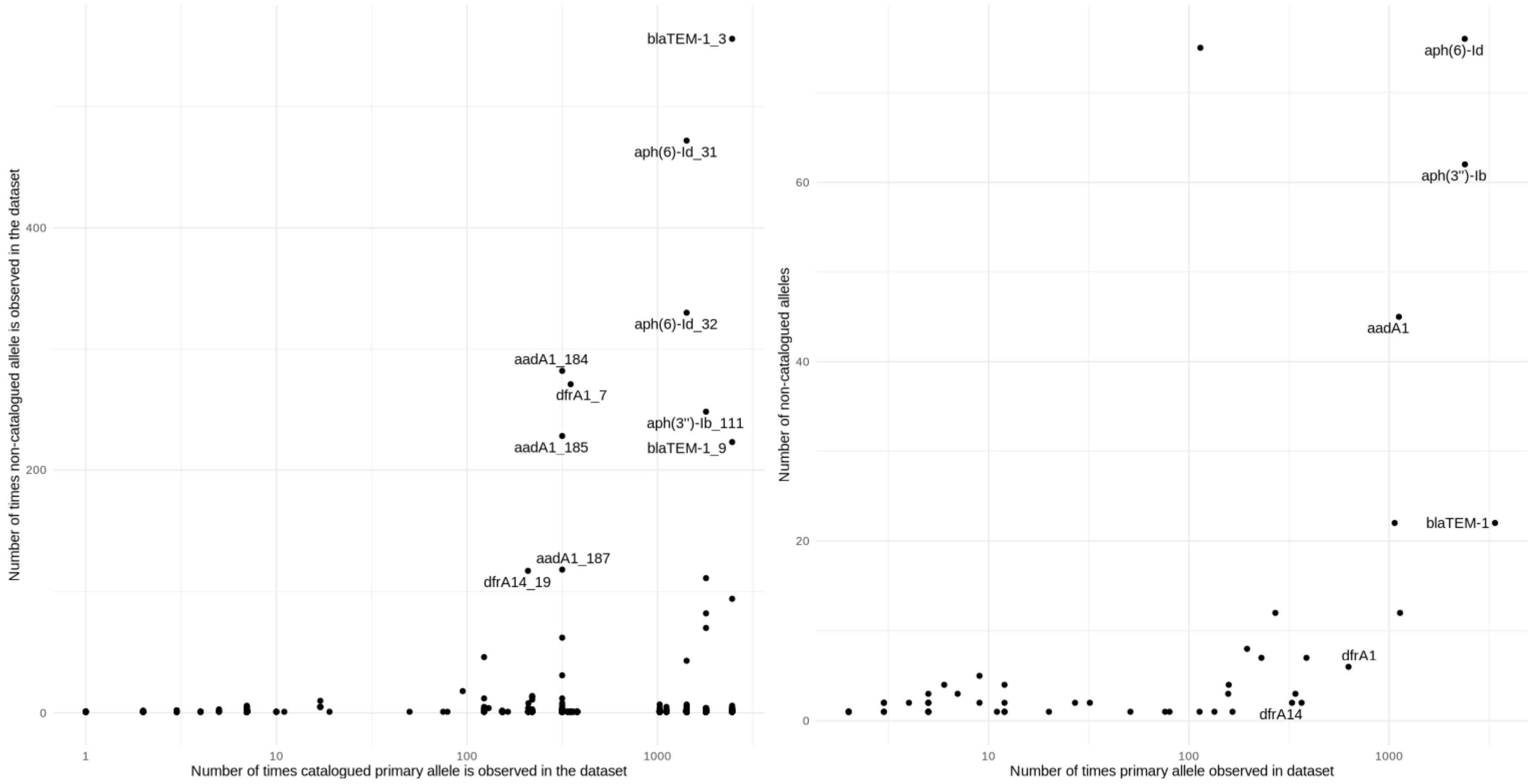

Figure S4 - non-catalogued alleles selected across the datasets in this study. In the left plot points represent uncatalogued alleles (non 100% nucleotide matches of ARGs in the AMRFinder database) whereas in the right they represent cataloged alleles at 100% nucleotide match to the AMRFinder database reference.

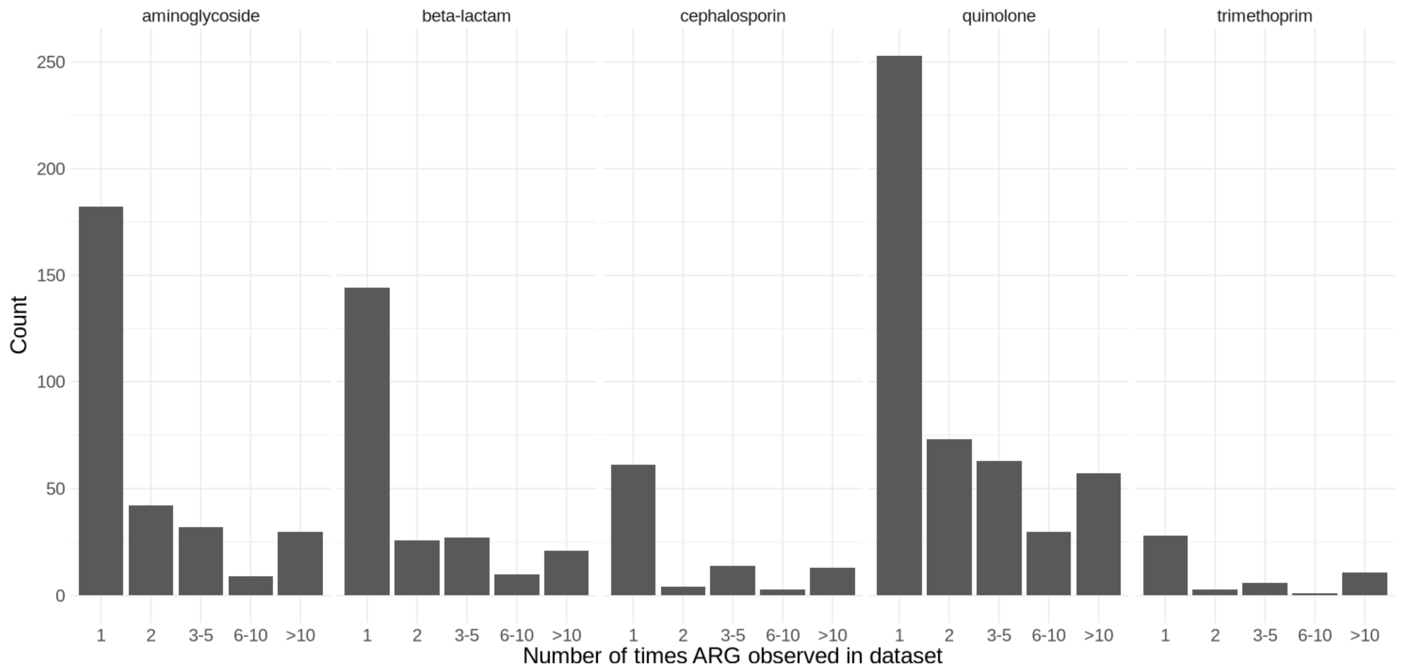

Figure S5 - Histograms of the number of times each unique ARG is observed in the dataset, stratified by class of antibiotic.

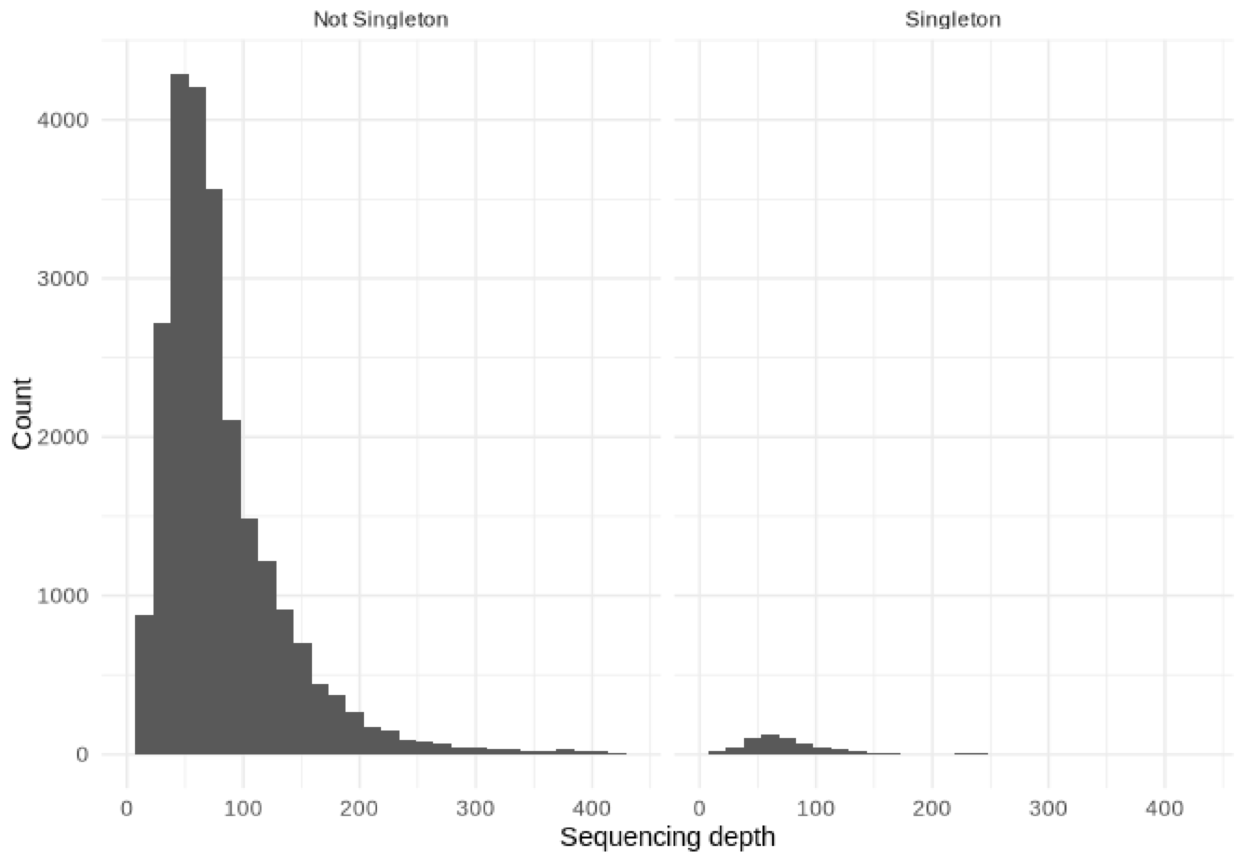

Figure S6 - distribution of median sequencing read depths for singleton vs non-singleton ARGs.

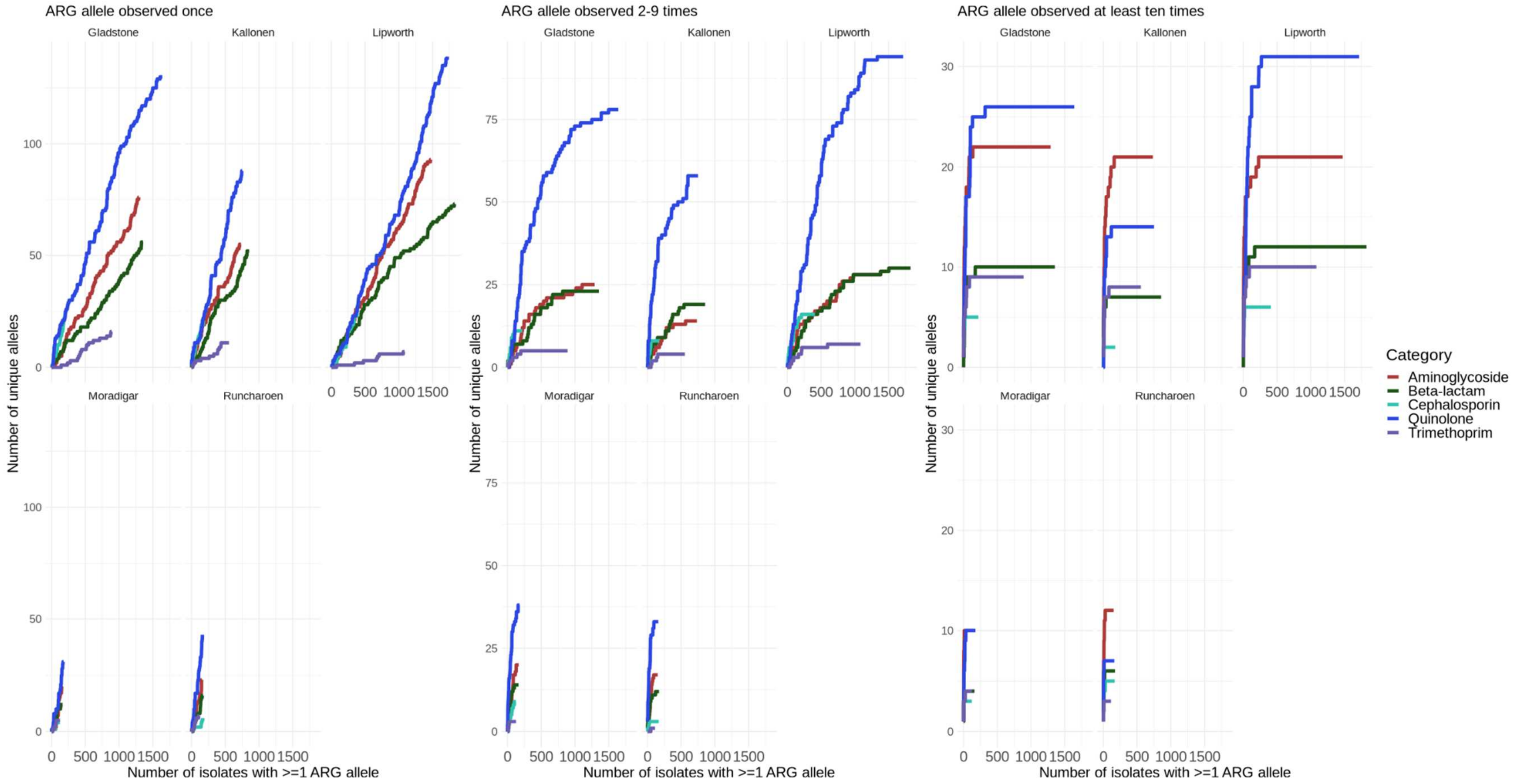

Figure S7- Simulated accumulation curves to show the relationship between the total number of unique ARGs observed and the number of isolates with at least 1 ARG of the antibiotic class denoted by the colour of the line in the dataset, stratified by the study in which the isolates were originally collected in.

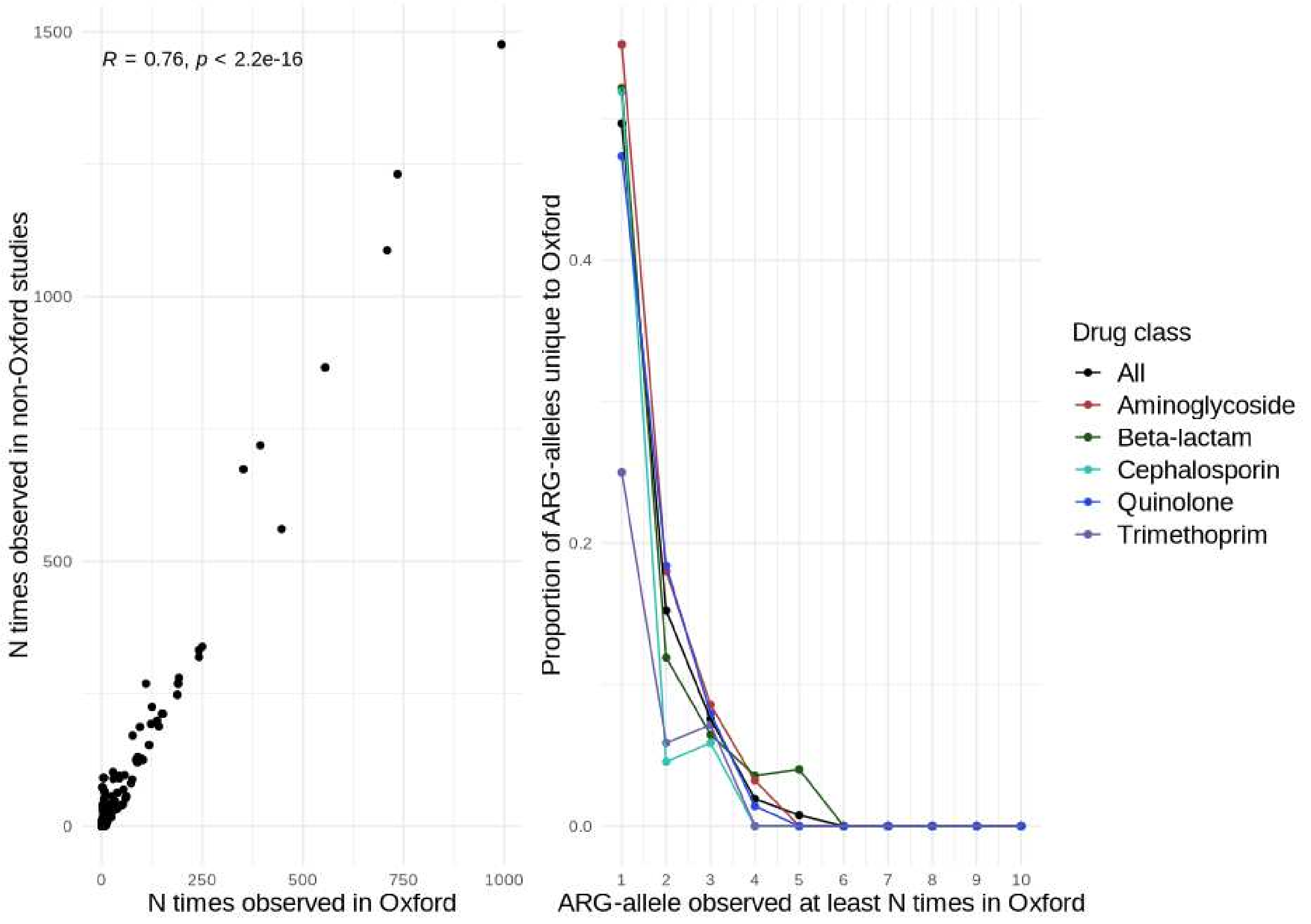

Figure S8 - Left - Correlation between the number of times an allele was observed in Oxford (N=2653 in total) and the rest of the dataset. Right - relationship between the proportion of ARG-alleles which are unique to the Oxford dataset (y-axis) compared to the number of times they are observed in the Oxford dataset (x-axis)

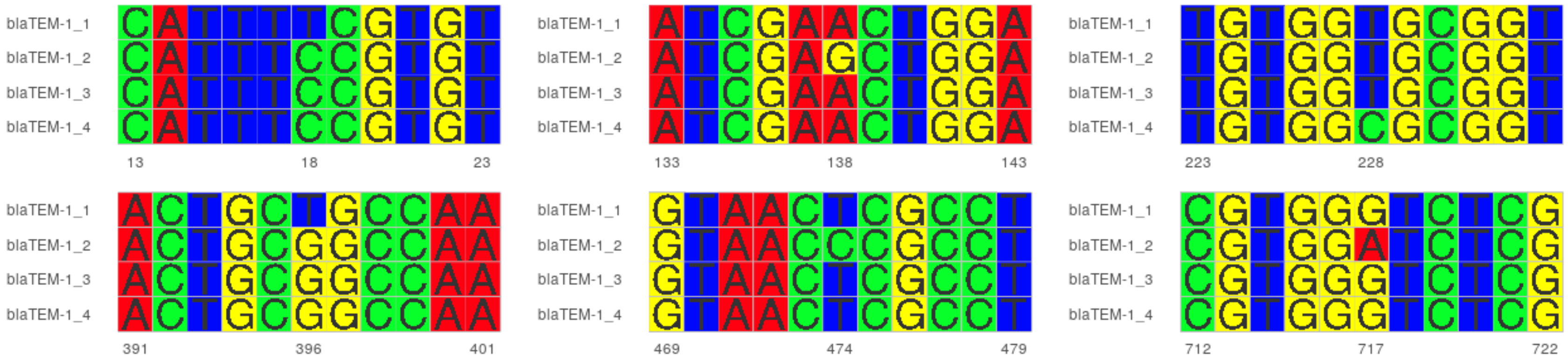

Figure S9 - Multiple sequence alignments highlighting the six synonymous polymorphic sites identified in bla_{TEM-1} that distinguish the four most common alleles of this gene identified in this study.

Supplementary References

1 Oksanen J, Blanchet FG, Friendly M, *et al.* vegan: Community Ecology Package. 2019. https://CRAN.R-project.org/package=vegan.

2 Ondov BD, Treangen TJ, Melsted P, *et al.* Mash: fast genome and metagenome distance estimation using MinHash. *Genome Biol* 2016; **17**: 132.

3 R Core Team. R: A Language and Environment for Statistical Computing. 2021. https://www.R-project.org/.

4 Gladstone RA, McNally A, Pöntinen AK, *et al.* Emergence and dissemination of antimicrobial resistance in Escherichia coli causing bloodstream infections in Norway in 2002–17: a nationwide, longitudinal, microbial population genomic study. *The Lancet Microbe* 2021; published online May 10. DOI:10.1016/S2666-5247(21)00031-8.

5 Kallonen T, Brodrick HJ, Harris SR, *et al.* Systematic longitudinal survey of invasive Escherichia coli in England demonstrates a stable population structure only transiently disturbed by the emergence of ST131. *Genome Res* 2017; published online July 18. DOI:10.1101/gr.216606.116.

6 Runcharoen C, Moradigaravand D, Blane B, *et al.* Whole genome sequencing reveals high-resolution epidemiological links between clinical and environmental Klebsiella pneumoniae. *Genome Med* 2017; **9**: 6.

7 Lipworth SIW, Vihta KD, Chau K, *et al.* Ten year longitudinal molecular epidemiology study of Escherichia coli and Klebsiella species bloodstream infections in Oxfordshire, UK. *Genome Med* 2021.

8 Moradigaravand D, Palm M, Farewell A, Mustonen V, Warringer J, Parts L. Prediction of antibiotic resistance in Escherichia coli from large-scale pan-genome data. *PLoS Comput Biol* 2018; **14**: e1006258.
